# A review of the epidemiology of oral and pharyngeal carcinoma: update

**DOI:** 10.1186/1758-3284-4-1

**Published:** 2012-01-13

**Authors:** Daniel M Saman

**Affiliations:** 1 University of Kentucky College of Public Health, Epidemiology, Lexington, Kentucky, USA

**Keywords:** oral and pharyngeal cancers, epidemiology, oral health epidemiology, literature review

## Abstract

Oral and pharyngeal cancers are the sixth most common cancers internationally. In the United States, there are about 30,000 new cases of oral and pharyngeal cancers diagnosed each year. Furthermore, survival rates for oral and pharyngeal cancers have not significantly improved over the last three decades. This review examines the scientific literature surrounding the epidemiology of oral and pharyngeal cancers, including but not limited to risk factors, disparities, preventative factors, and the epidemiology in countries outside the United States. The literature review revealed that much of the research in this field has been focused on alcohol, tobacco, and their combined effects on oral and pharyngeal cancers. The literature on oral and pharyngeal cancer disparities among racial groups also appears to be growing. However, less literature is available on the influence of dietary factors on these cancers. Finally, effective interventions for the reduction of oral and pharyngeal cancers are discussed.

## Introduction

Oral and pharyngeal cancers (OPC) are considered an important part of the global burden of cancer, mainly due to the widespread use of tobacco and alcohol. In the United States, cancers of the oral cavity account for nearly 2.3% of cancers and also have a relatively low five-year survival rate [1]. There are also nearly 30,000 new cases of OPC diagnosed each year in the United States with about 8,000 people dying of these malignancies and evidence of little improvement in five-year survival rates from 1973-1998 [2-4]. In addition, OPC are among the most common (sixth most) cancers internationally [5,6]. Moreover, tobacco and alcohol use attribute significantly to the risk of developing these types of malignancies. The population-attributable risk of smoking and alcohol use for developing OPC has been estimated at 80% for males, 61% for females, and 74% in general [7].

Though much of the literature on OPC risk factors is dominated by tobacco studies, there have been several studies that have established the protective effects of a healthful diet. Certain protective factors against developing OPC include the consumption of coffee, vegetables, fruit, and dietary folate intake [8,9]. Other protective factors may include socioeconomic-based variables. The literature points to a differential distribution of OPC among minorities and other sub-populations. There seems to be a disproportional burden of OPC among certain sub-populations within and outside of the United States. For example, studies in the United States have shown disparities in OPC between Appalachian states and non-Appalachian states [1], low socioeconomic and higher socioeconomic status populations [10], black and white Americans [11], and males and females [12]. Disparities also exist in places outside of the United States, such as Taiwan [13]. Globally, significant differences in the incidence of OPC have been observed by country, with men in northern France and southern India having the highest OPC incidence rates when compared to 47 other countries [14].

This review examines the scientific literature surrounding OPC, including but not limited to OPC risk factors, OPC disparities, OPC preventative factors, and OPC in countries outside the United States.

### Literature review methodology

The methods for the inclusion of scientific articles in this review are liberal in nature. This review does not aim to be wholly comprehensive of all the literature on OPC, rather, it highlights the most relevant articles in the literature, and discusses the most important finding in each study. No exclusion criteria were applied when deciding which scientific articles would be included in this review. The search engines *PubMed *[15], and *Google Scholar *[16] were used to locate the most relevant articles pertaining to oral and pharyngeal cancers, regardless of year published. Thus, this review includes studies from 1988 to 2009. Furthermore, a wide time frame was used to determine whether later studies differed in their results than earlier ones, and to assess how OPC related research has changed over time. Keywords in these searches included 'oral' and 'pharyngeal' and 'cancers'. Other keyword searches included 'oral and pharyngeal cancers', 'OPC malignancies', 'OPC risk factors', and 'OPC disparities'. Table 1 presents a portion of the literature review on risk and protective factors for OPC.

**Table 1:** OPC literature review: risk and protective factors for OPC

Authors (Ref.)	Year Published	Study Location	Study Type	Cases	Control	Study Results
Rodriguez T et al. [8]	2004	Italy, Switzerland	Case-Control	137	298	Heavy Smokers OR = 20.7; Heavy drinkers OR = 4.9; High tobacco and alcohol OR = 48; High coffee consumption OR = 0.25; High vegetable consumption OR = 0.39; High fruit consumption OR = 0.73; High beta carotene consumption OR = 0.48
Pelucchi C, et al. [9]	2003	Italy, Switzerland	Case-Control	749	1772	Highest tertile of dietary folate intake OR = 0.53; Combined OR for low folate and high alcohol OR = 22.3
Greenberg RS, et al. [10]	1991	United States	Case-Control	762	837	Low percentage of years worked OR = 2.3
Day GL, et al. [11]	1993	United States	Case-Control	1065	1182	Heavy drinking among blacks OR = 17; Heavy drinking among whites OR = 9;
Cook MB, et al. [12]	2009	United States	Retrospective	-	-	Male-to-female incidence rate ratio hypopharynx = 4.13; Male-to-female incidence rate ratio oropharynx = 3.06
Ho PS, et al. [13]	2007	Taiwan	Retrospective	-	-	Highest age-standardized mortality rate for females of aboriginal groups = 3.76; Highest age standardized incidence rate for females of aboriginal groups = 2.18
Franseschi S, et al. [14]	2000	United States, Europe, Asia, Africa, Australia (49 areas worldwide)	Retrospective	-	-	Internationally, OPC highest for men in Bas Rhin, France (49.4/100,000 male incidence); For men in Americas and Australia, OPC highest for blacks in the United States (17.8/100,000)
Franseschi S, et al. [19]	1999	Italy, Switzerland	Case-Control	274 oral cancer; 364 pharyngeal cancer	1254	Oral cancer, ≥ 77 drinks/week, ≥ 25 cigarettes/day, OR = 228; Pharyngeal cancer, > 77 drinks/week, > 25 cigarettes/day, OR = 100
Fioretti F, et al. [20]	1999	Italy	Case-Control	42 (lifelong non-smokers	864 (lifelong non-smokers	OPC drinkers vs. non drinkers OR = 3.0; OPC drinking 35+ years vs. non drinking OR = 3.6; High butter intake OR = 2.7
Blot WJ, et al. [21]	1988	United States	Case-Control	1114	1268	Two or more packs cigarettes smoked/day and four or more drinks/day OR = 37.7; Males who smoked filters OR = 0.5
Varela-Lema L, et al. [22]	2009	Spain	Case-Control	92	230	Ever smokers OR = 27.7
Talamini R, et al. [23]	1990	Italy	Case-Control	27 non-smokers; 19 non-drinkers	572 non-smokers; 213 non-drinkers	Non-smokers, 14-55 vs. 0-13 alcoholic drinks/week OR = 1.5; Non-smokers, 56+ alcoholic drinks/week, OR = 2.2; Non-drinkers, < 15 cigarettes/day OR = 3.8; Non-drinkers, > 15 cigarettes/day OR = 12.9
Kabat GC, et al. [24]	1994	United States	Case-Control	1560	2948	Male current smokers, users of filter cigarettes OR = 0.5
Aune D, et al. [25]	2009	Uruguay	Case-Control	3539	2032	High red meat consumption OR = 3.65
Kune GA, et al. [26]	1993	Australia	Case-Control	41	398	Fiber intake OR = 0.29; Vitamin C > 745 mg/week OR = 0.39
Soler M, et al. [27]	2001	Italy	Case-Control	271 oral cancer; 327 pharyngeal cancer; 304 esophageal cancer	1950	Highest fiber intake OR = 0.40
Negri E, et al. [28]	2000	Italy, Switzerland	Case-Control	754	1775	Highest Vitamin C intake OR = 0.63
Lucenteforte E, et al. [29]	2009	Italy	Metanalysis (6 cohort, 40 case-control studies)	-	-	High vegetable consumption pooled RR = 0.52; High fruit consumption pooled RR = 0.55
Gridley G, et al. [30]	1992	United States	Case-Control	1114	1268	"Ever regularly used" vitamin E, OR = 0.5
Levi F, et al. [31]	1998	Switzerland	Case-Control	156	284	Highest tertile of egg consumption OR = 2.3; Red meat OR = 2.1; Pork and processed meat OR = 3.2; Highest tertile for milk consumption OR = 0.4; fish OR = 0.5; raw vegetables OR = 0.3; cooked vegetables OR = 0.1; citrus fruit OR = 0.4; other fruits OR = 0.2
Franceschi S, et al. [32]	1999	Italy	Case-Control	598	1491	Highest quintile coffee and tea OR = 0.6; Highest quintile white bread OR = 0.4; Highest quintile soups OR = 2.5; Highest quintile poultry OR = 0.6; Highest quintile fish OR = 0.6; Highest quintile eggs OR = 2.5; Highest quintile raw vegetables OR = 0.4; Highest quintile cooked vegetables OR = 0.5; Highest quintile citrus fruit OR = 0.5; Highest quintile cakes and desserts OR = 1.6
Zheng W, et al. [33]	1992	China	Case-Control	204	414	Highest tertile of fruit and vegetable consumption among men OR = 0.6
Lipworth L, et al. [34]	2009	Italy	Case-Control	804	2080	Vitamin D intake OR = 0.76; Heavy smokers and low dietary vitamin D intake OR = 10.4: Heavy drinkers and low dietary vitamin D intake OR = 8.5
Goldstein AM, et al. [35]	1994	United States	Case-Control	487	485	Odds for OPC increases for those whose sisters developed other cancers OR = 1.6
Huebner WW, et al. [36]	1992	United States	Case-Control	1114	1268	Male carpet installers OR = 7.7 (among carpet installers, 23 cases, 4 controls)
Goodwin WJ, et al. [37]	2008	United States	Retrospective	-	-	OPC greater in black than white populations; OPC survival lower in black than white populations
Morse DE, et al. [38]	2006	United States	Retrospective	-	-	OPC age adjusted incidence rates and mortality rates highest for black males; Mortality rates 82% higher for black males relative to white males
Tomar SL, et al. [39]	2004	United States	Retrospective	21481	-	Blacks had elevated hazard ratios compared to whites HR range: 1.20-1.53
Moore RJ, et al. [40]	2001	United States	Retrospective	909		African-Americans five-year survival rate of 27.6%; white patients five-year survival rate of 52.0%. African-American and white Americans less than 60 years of age had a survival rate of 29.2% and 60.9%, respectively.
Chen AY, et al. [41]	2007	United States	Retrospective	40487	-	Patients with advanced OPC more likely to be uninsured OR = 1.37

Also, unlike conditions such as the metabolic syndrome (MetS) where the definition is not wholly agreed upon or standardized [17], there was no evidence in the literature that this was the case for OPC. The International Classification of Diseases, Tenth Revision (ICD-10) defines OPC as any malignant neoplasm of lip, oral cavity (tongue, major salivary glands, gum, floor of mouth, other and unspecified parts of mouth), and pharynx (oropharynx, nasopharynx, and hypopharynx) [18].

### Risk factors

#### Tobacco and alcohol

Tobacco use is understood to be the most important risk factor for the development of OPC [7]. Much of the literature on OPC risk factors is focused specifically on tobacco use. For example, Rodriguez et al (2004) analyzed data from two case-control studies including 137 cases of OPC and showed that the multivariate odds ratios (OR) for having OPC for heavy smokers was 20.7 in young adults from Italy and Switzerland. Rodriguez et al found that the OR for heavy drinkers for OPC was 4.9 [8]. However, when the categories of heavy drinking and smoking were combined, an OR of over 48 was observed. The authors also found that tobacco accounted for 77% of OPC cases in the examined population, alcohol for 52%, low vegetable consumption for 52%, and the combination of the three for nearly 85% of all OPC cases [8].

A separate case-control study performed on Italian and Swiss men also found large risk increases for oral cancer (OR = 228) and pharyngeal cancer (OR = 100) for the highest level of drinking (≥ 77 drinks/week) and smoking (≥ 25 cigarettes/day) combined [19]. The authors of this study found there to be synergistic effects of smoking and alcohol consumption on OPC. The authors found interesting independent factors in that if alcohol consumption increased while smoking levels were stable, the increase in oral cancer would be greater than the increase in pharyngeal cancer. This was shown to be the case in this study because the authors explain that the ratios of ORs between oral cancer and pharyngeal cancer was about 2-times greater for oral cancer than for pharyngeal cancer for each combined level of smoking and drinking.

In addition, alcohol consumption has been shown to be associated with increased odds of OPC among never smokers. Fioretti et al (1999) examined 42 cases of OPC among never smokers and found that the major risk factor for OPC in never smokers was alcohol consumption, with an OR three-fold higher in drinkers than non-drinkers [20].

Another case-control study that confirmed a multiplicative synergism between smoking and alcohol consumption on OPC examined 1,114 cases and 1,268 controls. Similar results were found as the above studies in that among those that smoked two or more packs of cigarettes and four or more drinks per day, there was an observed 35-fold increase in the risk of OPC. The authors estimate that smoking and drinking combined account for about 75% of all OPC in the United States [21]. A similar study in a Spanish male population found that tobacco smoking was associated with OPC with an OR of 27.7 [22]. These studies combined confirm the magnitude that smoking and alcohol consumption have on OPC.

Moreover, a study examining the separate effects of alcohol in non-smokers and smoking in non-drinkers also found results similar to the above studies cited. This study was performed essentially to test the relative independence that smoking and alcohol consumption has on the development of OPC. The ORs were 1.5 for 14-55 versus 0-13 drinks per week, and 2.2 for 56 drinks or more in non-smokers. The ORs for non-drinkers who smoked were 3.8 for smokers of < 15 cigarettes per day and 12.9 for ≥ 15 cigarettes per day [23]. This is an important study in the literature because it establishes the independence that smoking and drinking have on the development of OPC.

Interestingly, two studies found that among male current smokers, those that used filter cigarettes had a reduced risk of oral cancer than those who used non-filter cigarettes [21,24]. However, little research has been performed on this topic, thus the results should be interpreted with caution.

#### Dietary risk factors

Less research has been focused on risk factors besides smoking and drinking and their relationship with OPC. However, there is some evidence that meat, vegetable, and vitamin intake may be related to OPC. A case-control study on meat consumption in Uruguay among nearly 4,000 cases showed a significant increase in the odds of having OPC (OR = 3.65, 95% CI 2.21-6.01) with a high intake of red meat [25]. Several studies have also found significantly protective effects of fiber intake on OPC (OR = 0.29, OR = 0.40) [26,27].

Dietary vitamin C consumption of > 745 mg/week was also shown to protect subjects from developing OPC (OR = 0.39) [26]. Another study also found that vitamin C had a similar protective effect (OR = 0.63) [28]. One study, however, found that vitamin C was protective of OPC, but noted that vitamin C's effect was difficult to separate from the effects of fruit and vegetables intake [29]. Vitamin E supplements (i.e., "ever regularly used") have also been found to be associated with a significantly reduced OPC risk with an adjusted OR of 0.5 (95%CI 0.4-0.6) [30]. Other studies have also shown that consumption of fruits (OR = 0.2), raw (OR = 0.3) and cooked vegetables (OR = 0.1), and fish (OR = 0.5) had inverse risks and protective effects on OPC [31,32]. Similar results were found among 414 cases in Shanghai, China, in that the risks of OPC development decreased with an increased intake of oranges, tangerines, other fruits, and some dark yellow vegetables, and white radishes. The authors found that men in the category with the highest intake of fruits and vegetables had an OR of about 0.5-0.7 when compared to men in the lowest group of fruit and vegetable consumption [33].

Vitamin D intake was also found to have an inverse risk on OPC (OR = 0.76) [34]. The same study found that the OR for OPC among heavy smokers with low dietary vitamin D intake was 10.4 (95%CI 6.9-15.5) and an OR of 8.5 (95%CI 5.7-12.5) among heavy alcohol drinkers with low dietary vitamin D intake. When looked at as a whole, these studies provide substantial evidence that dietary factors play a very important role in OPC development.

#### Other risk factors

Even fewer studies have examined factors besides tobacco, alcohol, and diet and the relation to OPC development. A familial-based study found non-significant odds ratios associated with any cancer in the family. However, a slightly elevated OR of 1.6 (95%CI 1.1-2.2) for OPC was found among family members whose sisters developed cancers [35]. This association, however, may be due to unaccounted confounders. Occupational risk factors have also been examined briefly in the literature. Huebner et al (1992) performed a population-based case-control study in four United States areas and found elevated OPC risks among male carpet installers (OR = 7.7, 95%CI 2.4-24.9) [36]. However, this study only looked at 23 cases and 4 controls, which presents a clear limitation in the power of the study.

### OPC disparities

#### Racial disparities

Though much of the literature regarding the risk factors of OPC is slightly dated, this is not the case for OPC disparities research. This type of research in the literature seems relatively new in nature. Much of the literature shows there to be differences in mortality, incidence, and survival among black and white males in the United States. For example, Goodwin et al (2008) shows there to be an unequal burden or disparity among black and white Americans with respect to OPC incidence and survival [37]. They argue that though the underlying causes of the differences may be unknown, the differences are likely to arise from several differing interplays and relationships among various components such as access to healthcare, quality of care, diet, cultural beliefs, etc. However, the authors argue for further prospective-cohort studies to more precisely explain the differences in OPC among black and white Americans [37]. Furthermore, Morse and Kerr (2006) found that although age-adjusted incidence rates and mortality rates declined for black and white males and females from 1975-2002, disparities still existed between black and white Americans. The authors found that age-adjusted mortality rates with black males were 82% higher than with white males [38].

Within a specific state, a study performed on OPC survival in Florida found that black Americans had higher hazard ratios (HR range: 1.20-1.53) than white Americans across all sites and stages [39]. An interesting finding of this study was that the differences in survival were not wholly because of differences in treatment. In another study, it was found that African-Americans had a significantly lower five-year survival rate of 27.6% (95%CI 19.9-38.3) than white patients with a survival rate of 52.0% (95%CI 48.7-55.6). The greatest racial disparity in survival in this study, interestingly, was found for patients less than 60 years of age. African-American and white Americans less than 60 years of age had a survival rate of 29.2% (95%CI 19.5-43.6) and 60.9% (95%CI 56.3-66.0), respectively [40]. One of the reasons this disparity may exist between black and white Americans is because of a stronger genetic interaction between smoking and alcohol consumption in black Americans [40].

#### Other OPC disparities

In addition, the impact of health insurance status on stage of diagnosis of OPC has been found to lead to a late-stage diagnosis. Schrage and Halpern (2007) found that patients with advanced OPC at diagnosis were more likely to be uninsured with an OR of 1.37 (95%CI 1.21-1.25) compared with patients who had private insurance. Similar results were found for patients who were covered by Medicaid, who presented with the largest tumors, and with the greatest degree of lymph node involvement. This study presents a diagnosis disparity based on insurance status. That is, being uninsured or on Medicaid increased the odds of presenting with more advanced and severe OPC, thus increasing the risk of lower survival [41] (see Table 1).

### Interventions

Several other studies have also found racial disparities for OPC among black and white Americans [42-44]. The recommendations made by these authors for reducing OPC disparities in incidence, mortality, and survival include early-stage diagnosis and an active role taken by dentists to reduce smoking and alcohol use among their patients. Morse and Kerr (2006) argue that dentists must aid in reducing OPC incidence and mortality by assisting patients in quitting smoking and eliminating alcohol abuse [38]. They also argue that relative survival can be improved through early detection of OPC. Horowitz et al (2000) surveyed dentists and found that most dentists (86%) did not conduct oral cancer examinations on edentulous patients [45]. This finding spurred the authors to conclude that dentists play a key role in the determination of early-stage OPC and that dentists must determine patients' risks for OPC. This study also pointed to a need for providing dentists with new educational updates on OPC prevention and detection [45].

A review paper by Truman et al (2002) on the evidence in the literature on interventions for OPC concluded there was insufficient evidence on the effectiveness of population-based interventions for early detection of OPC [46]. This study points to a gap in the literature regarding interventions on early detection of pre-cancers and OPC.

### Social concerns

One important social issue associated with OPC deals mainly with the disparity present between black and white Americans. Though this disparity may potentially be due to an unaccounted confounder, the burden of OPC mortality and lower survival is placed heavily on black Americans. Inability to pay for medical bills, increased rates of smoking and alcohol use, cultural beliefs, a late-stage diagnosis of OPC, etc, may be partly due to the lower survival rates and higher mortality rates experienced by black Americans with respect to OPC.

### Recommendation and expected outcomes

Improving the incidence, mortality, and survival rates of OPC requires a multi-tier structural approach that targets society, dentists, communities, and the individual. Dentists should encourage their patients to quit smoking and abusing alcohol. In addition, dentists could also be compensated for reducing smoking rates among their patient population; this would be a clear incentive for dentists to engage in their patient population. Silverman (2001) argues that efforts should be maximized to increase the early detection of localized lesions and pre-cancers, coupled with "aggressive" counseling in tobacco cessation and alcohol use for OPC outcomes to improve [2].

Reducing smoking rates through the enactment of a nation-wide smoking ban would also reduce OPC, given the highly interrelatedness of smoking and OPC. Reducing alcohol consumption should also be targeted. The number of alcohol outlets can be mitigated by making it available only in pharmacies. This policy can also be applied to cigarettes.

## Conclusions

This review revealed interesting information about the literature on OPC. There were several noticeable biases within the literature, however. Many of the research articles cited included European study populations. Populations in non-European countries must be examined to ensure the relative generalizability of the results. Also, most of these studies were case-control studies, and not prospective cohort studies. This presents a limitation in the generalizablitiy and causal inference one can make from these studies. However, that most of the studies found similar results highlight the strength of the association of several of the risk factors (e.g., tobacco and alcohol use, and diet) related to OPC. Future research study designs should be prospective cohorts with large sample sizes that firmly establish the risk factors associated with the development of OPC.

## List of abbreviations

OPC: oral and pharyngeal cancers; MetS: metabolic syndrome; ICD-10: International Classification of Diseases-tenth revision; OR: odds ratio; HR: hazard ratio.

## Competing interests

The author declares that he has no competing interests.

## Authors' contributions

DMS conceived the manuscript and wrote the entirety of the manuscript. All authors read and approved the final manuscript.

## References

[B1] Casto BC Sharma S Fisher JL Knobloch TJ Agrawal A Weghorst CM Oral cancer in Appalachia J Heath Care Poor Underserved 2009 20 274 85 10.1353/hpu.0.0097 19202262

[B2] Silverman S Demographics and occurrence of oral and pharyngeal cancers: the outcomes, the trends, the challenge J Am Dent Assoc 2001 132 7S 11S 11803655 10.14219/jada.archive.2001.0382

[B3] U.S. Department of Health and Human Services With understanding and improving health and objectives for improving health Healthy People 2010 2000 2 2 Washington, DC: U.S. Government Printing Office

[B4] Greenlee RT Hill-Haron B Murray T Thun M Cancer statistics, 2001 CA Cancer J Clin 2001 51 15 36 10.3322/canjclin.51.1.15 11577478

[B5] Dwivedi RC Kazi RA Agrawal N Nutting CM Clarke PM Kerawala CJ Rhys-Evans PH Harrington KJ Evaluation of speech outcomes following treatment of oral and pharyngeal cancers Cancer Treat Rev 2009 35 417 24 10.1016/j.ctrv.2009.04.013 19481871

[B6] Warnakulasuriya S Global epidemiology of oral and oropharyngeal cancer Oral Oncol 2009 45 309 16 10.1016/j.oraloncology.2008.06.002 18804401

[B7] Peterson PE Oral caner prevention and control-the approach of the World Health Organization Oral Oncol 2009 45 454 60 10.1016/j.oraloncology.2008.05.023 18804412

[B8] Rodriguez T Altieri A Chatenoud L Gallus S Bosetti C Negri E Franceschi S Levi F Talamini R La Vecchia C Risk factors for oral and pharyngeal cancer in young adults Oral Oncol 2004 40 207 13 10.1016/j.oraloncology.2003.08.014 14693246

[B9] Pelucchi C Tamani R Negri E Levi F Conti E Franceschi S La Vecchia C Folate intake and risk of oral and pharyngeal cancer Ann Oncol 2003 14 1677 81 10.1093/annonc/mdg448 14581278

[B10] Greenberg RS Huber MJ Clark SW Brockman JE Liff JM Schoenberg JB Austin DF Preston-Martin S Stemhagen A Winn DM The relation of socioeconomic status to oral and pharyngeal cancer Epidemiology 1991 2 194 200 10.1097/00001648-199105000-00006 2054401

[B11] Day GL Blot WJ Austin DF Bernstein L Greenberg RS Preston-Martin S Schoenberg JB Winn DM McLaughlin JK Fraumeni JF Jr Racial differences in risk of oral and pharyngeal cancer: alcohol, tobacco, and other determinants J Natl Cancer Inst 1993 85 465 73 10.1093/jnci/85.6.465 8445674

[B12] Cook MB Dawsey SM Freedman ND Inskip PD Wichner SM Quraishi SM Devesa SS McGlynn KA Sex disparities in cancer incidence by period and age Cancer Epidemiol Biomarkers Prev 2009 18 1174 82 10.1158/1055-9965.EPI-08-1118 19293308 PMC2793271

[B13] Ho PS Yang YH Shieh TY Chen CH Tsai CC Ko YC Ethnic differences in the occurrence of oropharyngeal cancer in Taiwan Public Health 2007 121 765 73 10.1016/j.puhe.2007.02.001 17499319

[B14] Franseschi S Bidoli E Herroro R Munoz N Comparison of cancers of the oral cavity and pharynx worldwide: etiological clues Oral Oncol 2000 36 106 15 10.1016/S1368-8375(99)00070-6 10889929

[B15] PubMed. National Center for Biotechnology Information, US National Library of Medicine 2009 At http://www.ncbi.nlm.nih.gov/pubmed/. Accessed October 10-November 10

[B16] Google Scholar 2009 At http://scholar.google.com/schhp?hl=en. Accessed October 29-November 10

[B17] Pratley RE Metabolic syndrome: why the controversy? Curr Diab Rep 2007 7 56 9 10.1007/s11892-007-0010-x 17348123

[B18] World Health Organisation International Classification of Diseases, Tenth Revision (ICD-10) 2010 http://apps.who.int/classifications/icd10/browse/2010/en. Accessed January 20

[B19] Franseschi S Levi F La Vecchia C Conti E Dal Maso L Barzan L Talamini R Comparison of the effect of smoking and alcohol drinking between oral and pharyngeal cancer Int J Cancer 1999 83 1 4 10449598 10.1002/(sici)1097-0215(19990924)83:1<1::aid-ijc1>3.0.co;2-8

[B20] Fioretti F Bosetti C Tavani A Franceschi S Vecchia CL Risk factors for oral and pharyngeal cancer in never smokers Oral Oncol 1999 35 375 8 10.1016/S1368-8375(98)00125-0 10645401

[B21] Blot WJ McLaughlin JK Winn DM Austin DF Greenberg RS Preston-Martin S Bernstein L Schoenberg JB Stemhagen A Fraumeni JF Smoking and drinking in relation to oral and pharyngeal cancer Cancer Res 1988 48 3282 7 3365707

[B22] Varela-Lema L Ruano-Ravina A Crespo MA Barros-Dios JM Tobacco consumption and oral and pharyngeal cancer in a Spanish male population Cancer Lett 2009 288 28 35 19615813 10.1016/j.canlet.2009.06.015

[B23] Talamini R Franceschi S Barra S Vecchia CL The role of alcohol in oral and pharyngeal cancer in non-smokers, and of tobacco in non-drinkers Int J Cancer 1990 46 391 3 10.1002/ijc.2910460310 2394506

[B24] Kabat GC Chang CJ Wynder EL The role of tobacco, alcohol use, and body mass index in oral and pharyngeal cancer Int J Epidemiol 1994 23 1137 44 10.1093/ije/23.6.1137 7721514

[B25] Aune D Stefani ED Ronco A Boffetta P Deneo-Pellegrini H Acosta G Mendilaharsu M Meat consumption and cancer risk: a case-control study in Uruguay Asian Pac J Cancer Prev 2009 10 429 36 19640186

[B26] Kune GA Kune S Field B Watson LF Cleland H Merenstein D Vitetta L Oral and pharyngeal cancer, diet, smoking, alcohol, and serum vitamin A and beta-carotene levels: a case-control study in men Nutr Cancer 1993 20 61 70 10.1080/01635589309514271 8415131

[B27] Soler M Bosetti C Franseschi S Negri E Zambon P Talamini R Conti E La Vecchia C Fiber intake and the risk of oral, pharyngeal and esophageal cancer Int J Cancer 2001 91 283 7 10.1002/1097-0215(200002)9999:9999<::AID-IJC1047>3.0.CO;2-I 11169948

[B28] Negri E Franceschi S Bosetti C Levi F Conti E Parpinel M La Vecchia C Selected micronutrients and oral and pharyngeal cancer Int J Cancer 2000 86 122 7 10.1002/(SICI)1097-0215(20000401)86:1<122::AID-IJC19>3.0.CO;2-2 10728605

[B29] Lucenteforte E Garavello W Bosetti C Vecchia CL Dietary factors and oral and pharyngeal cancer risk Oral Oncol 2009 45 461 7 10.1016/j.oraloncology.2008.09.002 18990606

[B30] Gridley G McLaughlin JK Block G Blot WJ Gluch M Fraumeni JF Vitamin supplement use and reduced risk of oral and pharyngeal cancer Am J Epidemiol 1992 135 1083 1092 1632421 10.1093/oxfordjournals.aje.a116208

[B31] Levi F Pasche C Vecchia CL Lucchini F Franceschi S Monnier P Food groups and risk of oral and pharyngeal cancer Int J Cancer 1998 77 705 9 10.1002/(SICI)1097-0215(19980831)77:5<705::AID-IJC8>3.0.CO;2-Z 9688303

[B32] Franceschi S Favero A Conti E Talamini R Volpe R Negri E Barzan L La Vecchia C Food groups, oils and butter, and cancer of the oral cavity and pharynx Br J Cancer 1999 80 614 20 10.1038/sj.bjc.6690400 10408875 PMC2362347

[B33] Zheng W Blot WJ Shu XO Diamond EL Gao YT Ji BT Fraumeni JF Jr Risk factors for oral and pharyngeal cancer in Shanghai, with emphasis on diet Cancer Epidemiol Biomarkers Prev 1992 1 441 8 1302555

[B34] Lipworth L Rossi M McLaughlin JK Negri E Talamini R Levi F Franceschi S La Vecchia C Dietary vitamin D and cancers of the oral cavity and esophagus Ann Oncol 2009 20 1576 81 10.1093/annonc/mdp036 19487490

[B35] Goldstein AM Blot WJ Greenberg RS Schoenberg JB Austin DF Preston-Martin S Winn DM Bernstein L McLaughlin JK Fraumeni JF Jr Familial risk in oral and pharyngeal cancer Eur J Cancer B Oral Oncol 1994 30B 319 22 7703800 10.1016/0964-1955(94)90032-9

[B36] Huebner WW Schoenberg JB Kelsey JL Wilcox HB McLaughlin JK Greenberg RS Preston-Martin S Austin DF Stemhagen A Blot WJ Oral and pharyngeal cancer and occupation: a case-control study Epidemiology 1992 3 300 9 10.1097/00001648-199207000-00005 1637894

[B37] Goodwin JW Thomas GR Parker DF Joseph D Levis S Franzmann E Anello C Hu JJ Unequal burden of head and neck cancer in the United States Head Neck 2008 30 358 71 10.1002/hed.20710 17972309

[B38] Morse DE Kerr RA Disparities in oral and pharyngeal cancer incidence, mortality and survival among black and white Americans J Am Dent Assoc 2006 137 203 12 16521387 10.14219/jada.archive.2006.0146 PMC1398075

[B39] Tomar SL Loree M Logan H Racial differences in oral and pharyngeal cancer treatment and survival in Florida Cancer Causes Control 2004 15 601 9 15280639 10.1023/B:CACO.0000036166.21056.f9

[B40] Moore RJ Doherty DA DO K Chamberlain RM Khuri FR Racial disparity in survival of patients with squamous cell carcinoma of oral cavity and pharynx Ethn Health 2001 6 165 77 10.1080/13557850120078099 11696928

[B41] Chen AY Schrag NM Halpern MT Ward EM The impact of health insurance status on stage at diagnosis of oropharyngeal cancer Cancer 2007 110 395 402 10.1002/cncr.22788 17562558

[B42] Shiboski CH Schmidt BL Jordan RC Racial disparity in stage at diagnosis and survival among adults with oral cancer in the US Community Dent Oral Epidemiol 2007 35 233 40 10.1111/j.0301-5661.2007.00334.x 17518970

[B43] Shavers VL Harlan LC Winn D Davis WW Racial/ethnic patterns of care for cancers of the oral cavity, pharynx, larynx, sinuses, and salivary glands Cancer Metastasis Rev 2003 22 25 38 10.1023/A:1022255800411 12716034

[B44] Miller C Henry R Rayens M Disparities in risk of and survival from oropharyngeal squamous cell carcinoma Oral Surg Oral Med Oral Pathol Oral Radiol Endod 2003 95 570 5 10.1067/moe.2003.108 12738948

[B45] Horowitz AM Drury TF Goodman HS Yellowitz JA Oral pharyngeal cancer prevention and early detection: dentists' opinions and practices J Am Dent Assoc 2000 131 453 62 10770007 10.14219/jada.archive.2000.0201

[B46] Truman BI Gooch BF Sulemana I Gift HC Horowitz AM Evans CA Griffin SO Carande-Kulis VG Reviews of evidence on interventions to prevent dental caries, oral and pharyngeal cancers, and sports-related craniofacial injuries Am J Prev Med 2002 23 21 54 10.1016/S0749-3797(02)00449-X 12091093

